# Quality of Life of People Living with HIV in Australia: The Role of Stigma, Social Disconnection and Mental Health

**DOI:** 10.1007/s10461-022-03790-7

**Published:** 2022-07-13

**Authors:** Carley J. Mendonca, Toby R. O. Newton-John, Dion M. Alperstein, Kim Begley, Ruth M. Hennessy, Shiraze M. Bulsara

**Affiliations:** 1 Clinical Psychology, Graduate School of Health, University of Technology Sydney (UTS), Ultimo, NSW Australia; 2 The Albion Centre, 150 Albion Street, Surry Hills, NSW 2010 Australia

**Keywords:** Stigma, Discrimination, Quality of life, Social connectedness, Mental health, HIV, QoL

## Abstract

HIV is a manageable chronic illness, due to advances in biomedical management. However, many people living with HIV (PLHIV) continue to experience psychosocial challenges, which have been associated with poorer quality of life (QoL). This study aimed to explore how psychosocial factors contributed to the QoL of PLHIV in Australia; specifically, the relationship between HIV-related stigma, social connectedness, mental health, and QoL. Participants were 122 PLHIV attending The Albion Centre (a tertiary HIV clinic in Sydney, Australia), who completed questionnaires which measured HIV-related stigma, social support, mental health symptomology and QoL. Results indicated that HIV-related stigma predicted poorer QoL, as did mental health symptomology. Conversely, social connectedness improved QoL. Additionally, social connectedness was found to mediate the relationship between HIV-related stigma and QoL, whereas the hypothesized moderating role of mental health symptomology on this model was not significant. These findings provide insight into the impact of psychosocial factors on QoL, offering practitioners various points of clinical intervention.

## Introduction

Antiretroviral therapy (ART) has transformed the trajectory of Human Immunodeficiency Virus (HIV) from an acute, and often terminal, illness into a manageable chronic illness. This has allowed many people living with HIV (PLHIV) to experience healthy lives [1], with life expectancy approaching that of the general population [2]. The effectiveness of biomedical management allows clinical research and practice to broaden its scope and explore issues pertaining to psychosocial factors, such as quality of life (QoL). Research suggests that despite the success of HIV treatments, several psychosocial challenges remain and generally diminish QoL [3, 4]. In particular, HIV-related stigma/discrimination, social disconnection, and mental health difficulties remain prevalent among this population and have harmful effects on QoL [5, 6]. Further understanding of the relationship between factors associated with poorer QoL among PLHIV is important for the development of effective strategies to support this population [7].

### Quality of Life

The 2014 Joint United Nations Programme on HIV/AIDS (UNAIDS) global ‘90–90–90’ target for HIV management [8], particularly achieving viral suppression, is traditionally used to measure treatment success and has a clear biomedical focus [9, 10]. These targets are progressively being achieved globally; in Australia, HIV has largely been controlled through ART with recent estimates suggesting Australia’s Cascade is currently 90–92–97 [11, 12]. Despite this biomedical success, QoL among many PLHIV remains poor [13, 14] and thus a more holistic model encompassing QoL is considered integral to treatment success.

QoL is multifaceted and impacted by the complex interplay between a range of physical, psychological, social, and environmental factors [12], and is *“not merely the presence or absence of disease”* [12, p. 2]*.* The current medical targets fail to address psychosocial comorbidities and consequently cannot optimize QoL. Research demonstrates that although ART improves QoL for PLHIV, the residual impact of psychosocial comorbidities means PLHIV still experience poorer QoL than the general population, regardless of effective medical interventions [6, 15–17]. Additionally, QoL amongst PLHIV is more closely linked to psychosocial factors than physiological or medical aspects of HIV [5, 18]. Consequently, understanding the psychosocial determinants of QoL is vital.

### Recognizing Psychosocial Comorbidities

Recent literature has recognized the importance of QoL, advocating for the addition of a ‘fourth-90’ to the UNAIDS ‘90–90–90’ targets; that is, Health Related Quality of Life (HRQoL) [19]. HRQoL is a multidimensional construct encompassing physical, psychological, and social domains of health [19, 20]. HRQoL can be conceptualized as the aspects of QoL which impact/are impacted by health factors, distinct from QoL which is much broader [21]. HRQoL focuses on *living well* with HIV long-term, surpassing VL suppression as the primary goal [6], and captures the needs of PLHIV who have achieved VL suppression yet continue to face psychosocial challenges which diminish QoL.

While HRQoL issues are important in their own right, research has also shown that these psychosocial comorbidities (particularly HIV-related stigma) impede capacity to engage in medical care; that is, HIV-testing, treatment uptake and medication adherence [22–25]. Therefore, although VL suppression is achieved through ART, psychosocial comorbidities can disrupt adherence and persist as barriers to sustained biomedical management [26]. Consequently, addressing psychosocial factors and optimizing QoL is instrumental in augmenting HIV management [18].

### HIV-Related Stigma/Discrimination

It is widely recognized that HIV-related stigma remains prevalent, negatively impacting QoL amongst PLHIV [4, 23, 24, 27, 28]. HIV-related stigma reflects the devalued status that society attributes to PLHIV, encompassing negative beliefs, feelings and attitudes [29, 30], and often manifests as discrimination and unjust treatment. Additionally, HIV-related stigma can be experienced as internalized stigma, considered to be negative feelings and beliefs about HIV applied to oneself [24, 31], which can damage self-esteem and psychological QoL [31–33] and can prevent PLHIV from satisfying social and medical needs [33, 34]. In Australia, there are high rates of transmission among men who have sex with men (MSM; [18]). Other populations deemed ‘at risk’ of HIV transmission are people who inject drugs, transgender people, people who engage in sex work, prisoners, and other incarcerated people [35]. Consequently, researchers suggest that PLHIV can experience multi-layered stigma, relating not only to their HIV status but also their membership of a minority group [22, 28, 32]. This has been associated with compounding deleterious effects on QoL [22, 29, 36].

### Social Disconnection

Social connection is an individual’s perception of belonging to a social network of satisfactory support, affection, and mutual aid [14]. It can be considered as structural (i.e., the size of the network, or *quantity* of connections) or functional (i.e., the perceived *quality* of connections; [37–39]). The literature has established a strong relationship between greater perceived social connectedness and greater QoL among PLHIV [18, 40, 41], in addition to documenting the protective and stress-buffering effects of social support [1, 42–44]. However, research also suggests that in general, PLHIV report social disconnection [18] and poor social support satisfaction [1, 45, 46].

The association between HIV-related stigma and social disconnection is well documented [42, 43, 46]. HIV-related stigma has been strongly associated with concerns about disclosing HIV-status, and subsequent non-disclosure [37, 47]. The degree of disclosure has been positively associated with social support satisfaction [45], with disclosure concerns, and subsequent non-disclosure, associated with poorer perceived social support and QoL [1, 48–51]. Similarly, disclosure can result in negative reactions and reduced social support due to stigma [52, 53], and ultimately poorer QoL [15, 54]. Conversely, social support has been shown to mediate the detrimental effects of stigma on QoL and depression [43]. Collectively, the literature demonstrates a complex relationship between HIV-related stigma, social connection and QoL.

### Mental Health Difficulties

Research indicates that mental health disorders are more prevalent among PLHIV compared to the general population [42, 55–57], with a mental health diagnosis associated with poorer QoL [3, 18]. This may be due to the unique psychosocial challenges faced by PLHIV, such as disclosure concerns, disclosure decision making, and changes to social networks following diagnosis [1, 42, 44], which make these individuals particularly vulnerable to psychological distress [3]. There is also some suggestion that those with a pre-existing psychiatric conditions may be more vulnerable to contracting HIV as a result of sexual risk-taking [58], a pathway which also likely impacts QoL.

The literature also points to a complex relationship between stigma and mental health [42, 50], where stigma and discrimination can create harmful social environments which may detrimentally impact mental health [22]. Consequently, stigmatized minority groups are likely at greater risk of developing mental health disorders [59], which can impact QoL. Given HIV disproportionately affects traditionally marginalized groups, compounding stigma sources place PLHIV at an elevated risk of developing mental health concerns [22, 28, 59].

Mental illness is generally associated with poorer perceived social connectedness, with social withdrawal a transdiagnostic symptom of many psychiatric disorders [60], suggesting mental health difficulties may exacerbate the relationship between stigma and social disconnection referred to earlier [48, 50, 52, 54]. The literature to date considers the impact of these variables on QoL, though the specific relationship between them has so far not been investigated.

### The Present Study

Much of the existing literature regarding the factors associated with QoL has been conducted internationally. Psychosocial factors, including stigma, differ according to sociocultural contexts [27]. Therefore, the international literature may not accurately reflect the experiences of PLHIV in Australia, and research is required to identify whether international trends are reflected in an Australian cohort given approximately 80% of PLHIV in Australia live in urban regions that are generally well resourced [18]. Moreover, the existing literature primarily explores the impact of psychosocial variables on QoL in isolation, rather than considering their impact comorbidly.

The present study aims to explore the relationships between factors which impact QoL in a well-resourced, urban context. Although the inclusion of the ‘fourth-90’ focuses on HRQoL, the present study sought to investigate the broader concept of QoL. This allowed the inclusion of sociodemographic variables, which likely impact functioning and QoL, to be included in addition to health-specific factors, and the use of a QoL scale which has been empirically validated with an Australian sample of PLHIV. It is also consistent with the current New South Wales (NSW) and national Australian HIV Strategies, which specifically include a QoL target [35, 61]. The study explores the relationships between psychosocial factors and the subsequent impact of these relationships on QoL. More specifically, it is hypothesized that HIV-related stigma, social connectedness, and mental health will predict QoL. It is also hypothesized that social connectedness will mediate the relationship between HIV-related stigma and QoL. Additionally, it is expected that mental health will moderate the mediation between HIV-related stigma and social connectedness to impact QoL.

## Methods

### Participants

Participants were a sample of 122 patients recruited from The Albion Centre (Albion), a multidisciplinary HIV treatment centre in Sydney, Australia. This sample represents approximately 10% of PLHIV accessing Albion for medical HIV care. Participation was voluntary for clients over 18 years-old, proficient in English language, living with HIV and attending Albion for HIV specialist medical care. Questionnaires could be completed online or on paper. One participant was excluded due to missing data.

### Measures

The PozQoL is a 13-item self-report scale specifically designed to assess QoL for PLHIV [13]. It explores QoL across four domains: health concerns, psychological, social, and functional. Each domain is measured by three or four items, rated along a five-point Likert scale ranging from ‘not at all’ (1) to ‘extremely’ (5). Higher scores indicate higher QoL. The PozQoL has been empirically validated within an adult Australian sample of PLHIV, demonstrating good reliability (Cronbach α = 0.95) and validity [13, 60, 62], and identifies specific cut-offs for qualitative appraisals of QoL (Low, Moderate, High, Very High).

The Scale of Perceived Social Support in HIV (PSS-HIV) is a 12-item self-report measure that evaluates perceptions of social support among PLHIV [38]. It assesses social support satisfaction through exploring the quality, not quantity, of contacts [63]. The PSS-HIV comprises three sub-scales which capture different levels-of-need within social support [38]. This includes belonging (two-items); esteem (four-items); and self-development (six-items). Each item is rated on a five-point Likert scale, ranging from ‘strongly disagree’ (1) to ‘strongly agree’ (5). Scores range from 12 to 60. Higher scores indicate higher perceived social support. The PSS-HIV has been validated among PLHIV, demonstrating good reliability (Cronbach α = 0.91) and validity [38, 64].

The HIV Stigma Scale-Short Form (HSS-SF) is a 12-item self-report measure that explores perceptions of HIV-related stigma among PLHIV, in the domains of personalized stigma, disclosure concerns, concerns about public attitudes, and negative self-image [65]. Each domain is measured by three items rated on a four-point Likert scale, ranging from ‘strongly disagree’ (1) to ‘strongly agree’ (4) (6). Scores range from 12 to 48. Higher scores indicate higher perceived HIV-related stigma. The HSS-SF has demonstrated good reliability (Cronbach α > 0.7 for all domains) and validity within HIV populations [65, 66].

The Diagnostic and Statistical Manual, 5th Ed (DSM-5) Level 1 Cross-Cutting Symptom Measure-Adult (DSM-XC) is a psychiatric self-report clinical screening tool [67]. The DSM-XC comprises 23-items that assess the symptomology of 13 psychiatric domains. Each item is rated on a five-point Likert scale, ranging from ‘none’ (0) to ‘severe’ (4), which aims to assess the presence and severity of specific symptoms in line with respective DSM-5 criterion [67]. Higher scores indicate a higher degree of mental health symptomology (i.e., poorer mental health). The DSM-XC is typically used for clinical purposes; however, it has demonstrated utility in research settings [68–71]. The DSM-XC was selected as it screens a broad range of mental health disorders, facilitating more meaningful information compared to other measures which are limited to fewer psychiatric categories [70]. Notably, substances listed in Question 23 use US drug names and were therefore adapted to suit an Australian cohort (i.e., ‘Vicodin’ changed to ‘opioid/codeine-based medications’; ‘Adderall’ changed to ‘Dexamphetamine’).

### Procedure

A research advisory group, comprising researchers and clinicians in the HIV sector as well as PLHIV peers, were involved in the design of the study. Ethics approval was obtained from the University of Technology Sydney (UTS; HREC ETH21-5827) and South Eastern Sydney Local Health District (SESLHD; HREC 2020/ETH01434). Recruitment strategies included waiting room flyers, clinician recruitment, and SMS recruitment due to COVID-19 limitations. Data collection occurred from February 2021 until September 2021. Participation involved completing measures on Qualtrics [72], a secure questionnaire platform regularly used in research settings. Participants could elect to complete the questionnaires online (via a QR-code or URL) or on paper. Additionally, consent was given by participants to access their medical record for information, such as VL and illness duration. Participation took approximately 25 min, and participants were provided a $25 grocery voucher for their time.

### Statistical Analysis

Bivariate Pearson’s correlation analyses were conducted to explore potential relationships between the variables. A multiple linear regression analysis was conducted to explore hypothesis 1 and ascertain whether the variables of interest predicted the dependent variable (QoL). Exploratory analyses were conducted using the SPSS v.27 (SPSS v.27, Armonk, NY, USA) ‘PROCESS’ macro (v. 3.5.3; [73]), to explore whether specific relationships between the variables predicted the dependent variable. Hypothesis 2 was tested through a mediation analysis (PROCESS macro model 4), exploring the indirect effects of stigma (independent variable) on QoL (dependent variable), through social connectedness (mediating variable). Hypothesis 3 was tested through a moderated mediation analysis (PROCESS macro model 7), exploring conditional indirect effects. That is, whether the relationship between stigma (independent variable) and social connectedness (mediating variable) varied at different levels of mental health symptomology (moderating variable), which in turn effects QoL (dependent variable).

## Results

The assumptions of a multiple regression analysis were tested and satisfied using SPSS v.27. This included multicollinearity, which was found to be within acceptable limits (no VIF value greater than 2 for any study variable), and normality (as assessed using histograms and normal Q–Q plots). Descriptive statistics provided information regarding demographic and independent variables. Items on measures were recoded, and subscales/totals summed, according to their respective instructions.

### Demographic Information

Demographic information is described in Table 1. Categorical variable groups were collapsed according to the number in each category, if the merge made conceptual sense. For example, ‘Transgender’ and ‘Other’ were collapsed into one category because of the low numbers in each cell. The mean age of participants was 50.32 years (*SD* = 12.85; range 27–77 years). The sample predominantly comprized male participants (*n* = 107 [87.7%]; Table 1), who identified as gay/queer (Table 1), consistent with the HIV-epidemic profile in Australia. The mean illness duration among participants was 16.29 years (*SD* = 10.58, range 0.5–38 years). Regarding participants’ VL, there were *n* = 4 missing; *n* = 117 undetectable VL; and *n* = 1 detectable VL.

**Table 1:** Demographic information

Variable	n (%)
Gender	
Male	107 (87.7)
Female	10 (8.2)
Transgender/other/non-disclosed	5 (4.1)
Sexual orientation	
Gay/queer	96 (78.7)
Heterosexual	14 (11.5)
Bisexual/other/non-disclosed	12 (9.8)
Relationship status	
Single	72 (59)
Married/de-facto/partnered	47 (38.5)
Casual/other	3 (2.5)
Income level	
$0–37,000	66 (54.1)
$37,001–90,000	41 (33.6)
$90,001 +	15 (12.3)
Highest education level	
High School	35 (28.7)
TAFE/College	32 (26.2)
University	55 (45.1)
Housing status	
Own home/rent	85 (69.7)
Department of housing/boarding/homeless/other	37 (30.3)

### Descriptive Statistics

Descriptive statistics for questionnaires to measure HIV-related stigma (HSS-SF- HIV), perceived social support (PSS-HIV) and mental health symptomology measures (DSM-XC) are shown in Table 2.

**Table 2:** Descriptive statistics

Measures	Mean	*SD*	Minimum	Maximum
PSS_HIV^a^	42.19	9.27	17	60
HSS_HIV^b^	30.59	7.41	12	48
DSM-XC^c^	45.58	15.24	23	84

^a^Perceived social support (PSS-HIV)

^b^HIV-related stigma/discrimination (HSS-SF)

^c^Mental health symptomology (DSM-XC)

During the development of the PozQoL, the researchers noted a broadly even distribution across the qualitative markers of QoL [13, 74]. The PozQoL results in the present study reflect a similar distribution (Table 3).

**Table 3:** Thresholds for interpreting the PozQoL^a^ [73]

Mean	*SD*	Minimum	Maximum	QoL^a^			
				Low	Moderate	High	Very high
44.48	11.06	15	65	29 (23.5%)	30 (24.5%)	35 (28.6%)	28 (22.9%)

^a^QoL (PozQoL)

### Bivariate Pearson’s Correlations

Bivariate Pearson's correlations were conducted to assess potential associations between age, illness duration, QoL, HIV-related stigma, perceived social connectedness and mental health symptomology. Table 4 presents a correlation matrix of the primary study variables.

**Table 4:** Pearson correlation matrix (n = 122)

	Age	Illness Duration	PozQoL^a^	PSS-HIV^b^	HSS-SF^c^	DSM-XC^d^
Age	1	0.649*	0.190*	− 0.246**	− 0.173	− 0.202*
Illness duration	–	1	0.186*	− 0.130	− 0.240**	− 0.068
PozQoL	–	–	1	0.447**	− 0.662**	− 0.640**
PSS-HIV	–	–	–	1	− 0.277*	− 0.336**
HSS-SF	–	–	–	–	1	0.356**
DSM	–	–	–	–	–	1

^a^QoL (PozQoL)

^b^Perceived social support (PSS-HIV)

^c^HIV-related stigma/discrimination (HSS-SF)

^d^Mental health symptomology (DSM-XC)

*p < 0.05

**p < .001

### Multiple Linear Regression

A multiple regression analysis was conducted to predict QoL from age, illness duration, gender, income level, HIV-related stigma, perceived social disconnection and mental health symptomology. The respective reference groups (comprising the greatest number of participants) were ‘male’ and ‘$0–$37,000’. For the purpose of these analyses, income was used as a proxy for socio-economic status (SES), which has occurred in previous studies (e.g., [75]).

The multiple regression model 1 significantly predicted QoL, (*F*[6, 115] = 3.14, *p* 0.007*, adj. R*^2^ = 0.096*).* The independent variables included in model 1 explained 9.6% of the variance in QoL. The multiple regression model 2 significantly predicted QoL, (*F*[9, 112] = 25.68*, p* < 0.001*, adj. R*^2^ = 0.647), with the independent variables explaining 64.7% of the variance in QoL. The respective regression coefficients are outlined in Table 5.

**Table 5:** Multiple regression results for QoL (n = 122)

Analyses	Variables	^a^R^2^	F	B^b^	SE^c^	β^d^	CI 95%	
							LLCI	ULCI
Model 1		0.096	3.142					
	Age			0.151	0.099	0.176	− 0.046	0.347
	Illness duration			0.124	0.121	0.118	− 0.116	0.363
	Female			1.185	3.552	0.030	− 5.850	8.220
	Transgender/other/nondisclosed			− 0.885	4.895	− 0.016	− 10.582	8.811
	$37,001–90,000			6.891*	2.146	0.296	2.640	11.141
	$90,001 +			7.409*	3.076	0.221	1.316	13.502
Model 2		0.647	25.676					
	Age			0.053	0.067	0.062	− 0.078	0.185
	Illness duration			0.061	0.078	0.058	− 0.094	0.215
	Female			3.544	2.284	0.088	− 0.981	8.069
	Transgender/other/nondisclosed			− 3.033	3.113	− 0.055	− 9.201	3.134
	$37,001–90,000			1.699	1.465	0.073	− 1.204	4.602
	$90,001 +			1.278	2.064	0.038	− 2.811	5.366
	HIV-related stigma			− 0.685**	0.092	− 0.468	− 0.867	− 0.503
	Perceived social support			0.197*	0.083	0.164	0.033	0.361
	Mental health symptoms			− 0.279**	0.045	− 0.392	− 0.369	− 0.188

Model = “enter” method in SPSS statistics

^a^R^2^ = adjusted R-squared

^b^B = unstandardised regression coefficient

^c^SE = standard error of the coefficient

^d^β = standardised coefficient

*p < 0.05

**p < 0.001

### Mediation Analysis

**Fig. 1:**
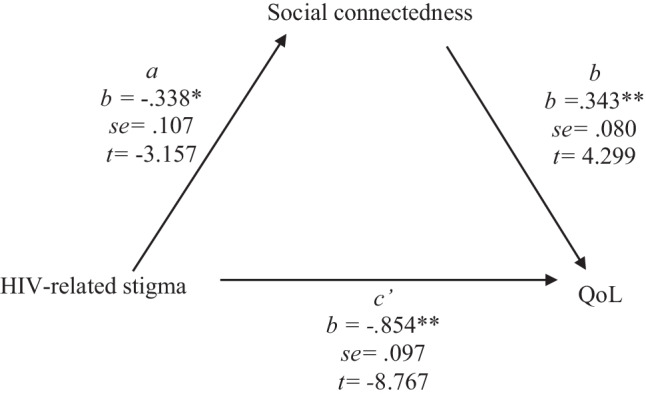
General pathway model describing social connectedness as a mediator for the relationship between HIV-related stigma and QoL, based on the PROCESS macro model 4 [73]. **p < 0.001, *p < 0.05

A mediation analysis (Fig. 1), using PROCESS macro model 4 [73], explored hypothesis 2. HIV-related stigma was negatively and significantly associated with perceived social connectedness (*t* = − 3.157, *p* < 0.002*, *95% CI − 0.5500: − 0.1260). Social connectedness was positively and significantly associated with QoL (*t* = 4.299,* p* < 0.001, 95% CI 0.1850; 0.5011) and HIV-related stigma was negatively and significantly associated with QoL (*t* = − 8.767, *p* < *0.0*01*, *95% CI − 1.0470; − 0.6612). The overall mediation model was supported, with a negative and significant indirect effect of HIV-related stigma on QoL, operating through social connectedness (*F*[1,120] = 93.727, *IE* = − 0.116*, *95% CI − *0.2*325; − 0.0354).

### Moderated Mediation Analysis

**Fig. 2:**
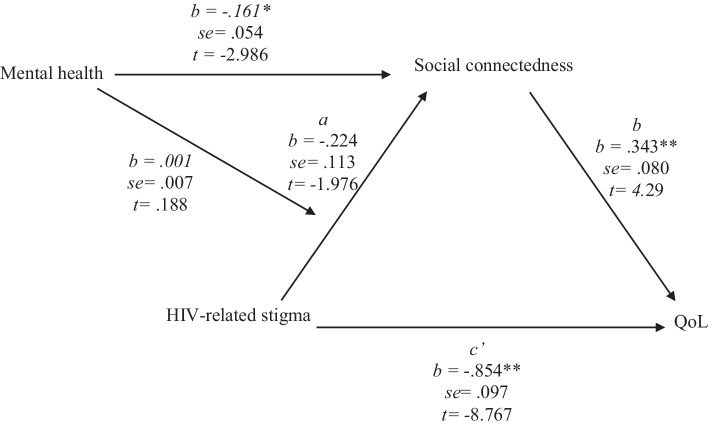
General pathway model describing mental health as a moderator for the mediated relationship between HIV-related stigma and QoL, based on the PROCESS macro model 7 [73]. **p < 0.001, *p < 0.05

A moderated mediation analysis, using PROCESS macro model 7 [73], was performed to explore hypothesis 3. In this model (Fig. 2), HIV-related stigma was negatively, yet non-significantly, associated with social connectedness (*t* = − 1.976*, p* 0.051*, *95% CI − 0.4479; 0.0005), while mental health was negatively and significantly associated with social connectedness (*t* = − 2.986*, p* < 0.003*, *95% CI − 0.2683; − 0.0543). The relationship between social connectedness and QoL was positive and significant (*t* = 4.299*, p* < 0.001*, *95% CI 0.1850; 0.5011). The relationship between HIV-related stigma and QoL was negative and significant (*t* = − 8.767,* p* < 0.001*, *95% CI − 1.0470; − 0.6612). The 95% confidence interval for the index of moderated mediation contained zero (*t* = 0.188*, p* < 0.851, 95% CI − 0118; 0.0142), suggesting there were no differences in the indirect effects at different levels of the moderator.

### Independent Samples T-test

An independent samples t-test compared the QoL mean scores for those obtained pre-COVID lockdown (n = 45) to those that were completed during lockdown (n = 77), and no significant difference was found (*t*[120] = − 0.89, *p* = 0.137).

## Discussion

While there is a significant evidence-base regarding the factors associated with QoL in PLHIV, there are relatively few Australian studies which have considered the variables comorbidly and explored how the relationships between them impact QoL. There was variability in QoL ratings among the present sample, consistent with previous research conducted among PLHIV in Australia [13, 18]. The results discussed below provide insight into this variability.

### Independent Effects of Stigma, Connectedness, and Mental Health Symptomology on QoL

This Australian-based study indicates HIV-related stigma, social disconnection, and mental health symptomology significantly predict QoL, consistent with international literature. In the present study, PLHIV who reported worse HIV-related stigma experienced poorer QoL. The construct of HIV-related stigma is multifaceted, covering internalized stigma, overt rejection and discrimination, and/or anticipated social stigma [24, 34], and each stigma-mechanism has been shown to negatively impact QoL [31, 35, 76, 77]. In the present study, although the HSS-SF only captured HIV-related stigma, the compounding deleterious effects of multi-layered stigma, including related to minority group status (e.g., MSM), may also contribute to this relationship [22, 29, 36].

As expected, a significant positive relationship was found between social connectedness and QoL, consistent with previous research [18, 40]. Social support has protective and stress buffering effects which not only improve QoL [41, 78], but may also protect against the effects of stigma and psychological distress [28, 40, 43, 44, 76] and promote coping following an HIV diagnosis [1]. In particular, the stress buffering effects of emotional support have been shown to improve QoL among PLHIV [41, 79]. Our findings suggests that the inverse may also be true, where social disconnection predicts poorer QoL.

Finally, increased mental health symptomology predicted poorer QoL in the current sample. It is well understood that the prevalence of mental health symptomatology in PLHIV is greater than the general population (e.g., [41]). More recently, the risk of composite mental illness, or comorbidity, has been found to be higher in PLHIV [80]. Given the established association between mental health and QoL (e.g., [18]), the present results are consistent within this context.

### The Relationship Between HIV-Related Stigma, Social Connectedness and QoL (Mediation Analysis)

While the evidence-base consistently reflects the associations discussed, relatively little research has explored the specific relationship between the variables, and their overall impact on QoL; understanding this, especially with an Australian cohort of PLHIV, is an important step in addressing these concerns. A mediation analysis was conducted to explore the relationship between psychosocial comorbidities and the subsequent impact of these relationships on QoL. Higher perceived HIV-related stigma was associated with poorer perceived social connectedness. Previous research suggests this may be due to the harmful effects of non-disclosure on social support satisfaction [47–49] and/or adverse reactions following disclosure [15, 53]. Recent surveillance data highlights the prevalence of HIV-related stigma in Australia, with more than half of respondents reporting the experience of stigma related to their HIV-status in the preceding 12-months, including from healthcare workers [18]. In the same survey, almost one third of participants noted that *‘almost nobody knows about my HIV’* [18], pointing to the link between HIV-related stigma and disclosure demonstrated in other Australian studies [27]; this ultimately has the potential to negatively impact social connectedness. In the present study greater perceived social connectedness was associated with greater QoL, and higher levels of HIV-related stigma were associated with poorer QoL [4, 23, 28]; as such a significant indirect effect of HIV-related stigma on QoL, operating through social connectedness, is indicated.

Several mechanisms are proposed to explain the link between HIV-related stigma, connectedness and QoL. The first pertains to the perceived quality of social support, a major component of social connectedness. Previous research has demonstrated that the degree of personal disclosure is positively associated with social support satisfaction [44]. However, stigma can influence how PLHIV navigate the challenging terrain of disclosure within relationships [1, 81]. Consequently, HIV-related stigma has been strongly associated with non-disclosure [37, 46], as a stigma-avoidant coping mechanism to preserve connectedness [82, 83]. Paradoxically, if personal disclosure enhances social connection, the effects of stigma and non-disclosure may create a barrier for connection with others. Consequently, stigma and non-disclosure have been previously associated with poorer perceived social support and QoL [1, 29, 47, 48, 77]. Additionally, some research suggests that stigma can be associated with unsupportive and adverse reactions following HIV disclosure [15, 45, 52, 53]. Therefore, stigma may enhance social disconnection which ultimately negatively impacts QoL.

Conversely, the protective and stress-buffering effects of social support may lessen the negative consequences of stigma on QoL. Researchers have found that social support can mitigate the harmful effects of stigma on QoL among PLHIV [42]. Similarly, the protective and stress buffering effects of social support have been well-documented and suggest greater perceived social connectedness may have the potential to reduce the harmful effects of stigma on QoL [1, 18, 28, 32, 39, 41, 43, 76, 78]. Our findings are consistent with international trends and indicate a complex relationship between HIV-related stigma, social connectedness and QoL in an Australian context, and further our understanding of these relationships.

### The Relationship Between HIV-Related Stigma, Social Connectedness, Mental Health and QoL (Moderated Mediation)

While the significant mediation model provides insight into the complex relationship between HIV-related stigma, social connectedness and QoL, it neglects the important role of mental health. A moderated mediation model was assessed to explore the impact of mental health on the relationship between stigma, connectedness and QoL. In the present study, mental health symptomology was significantly associated with poorer perceived social connectedness. This may be due to social withdrawal, a transdiagnostic feature of many psychological disorders [59]. It may also relate to the source of support; though the present study did not formally assess this, the importance of peer/community engagement for MSM and the associated positive impact on mental health symptomatology [84] cannot be discounted. It is, therefore, possible these results might reflect the absence of such support. Interestingly, HIV-related stigma was negatively, yet not significantly, associated with social connectedness in this model. This is inconsistent with the mediation results and previous research discussed above.

Contrary to hypothesis 3, the overall moderated mediation model was not supported suggesting worsening levels of mental health symptomology did not exacerbate the effects of stigma on connectedness to impact QoL. The Pearson’s correlation analysis suggests that mental health symptomology was highly correlated with all study variables. Additionally, previous research demonstrates a relationship between mental health and stigma [3, 59]; social connectedness [42]; and QoL [18]. The inclusion of mental health symptomatology to the model clearly changes the relationship between stigma and connectedness, and their impact on QoL, which suggests that mental health is implicated in this relationship; however, its effects were not captured by this model. Alternatively, it is possible that mental health symptomology is intrinsically related to stigma, connectedness, and mental health, such that the independent effects of mental health could not be determined statistically. To the authors’ knowledge, this is the first study to explore mental health as an independent variable in this way. Future research could explore the way in which mental health impacts QoL as a psychosocial comorbidity.

It is noteworthy that data collection partly occurred when the 2021 State government restrictions on face-to-face consults were in place, as part of the pandemic response to COVID-19, which may have impacted QoL. However, the independent samples t-test indicated no significant difference in QoL scores among PLHIV before and during the lockdown. This suggests that the findings discussed above reflect generalizable psychosocial challenges which impact QoL among PLHIV in Australia.

### Limitations

Due to the cross-sectional nature of this research design, true causality and mediation cannot be confirmed; as such, inferences should be drawn with caution. Moreover, utilizing the DSM-XC as a measure of mental health in the context of research bears limitations. The DSM-XC was used to measure mental health as it is brief, readily available and covers mental health symptomology across multiple psychiatric disorders. However, the measure is primarily used in a clinical setting to screen for mental health symptomology and is not diagnostic. Additionally, it is possible that the DSM-XC is too broad and does not account for the potential impact of symptoms—just their presence. The above limitations may have negated the study’s capacity to accurately capture mental health, which could explain the results of the moderated mediation analysis. Therefore, the results discussed above should be interpreted cautiously.

### Directions for Future Research

While the present study has contributed to our understanding of QoL in an Australian sample, the model explained 64.7% of the variance which suggests other factors which were not captured and should be the focus of future research. The present findings suggest that psychosocial comorbidities impact QoL in a complex way. Previous research demonstrates that mental health remains prevalent among PLHIV [55, 57, 79], and the results suggest that mental health is strongly correlated with all study variables and poorer QoL. However, the moderated mediation analysis suggests that mental health does not interact with psychosocial comorbidities to impact QoL as initially predicted. Therefore, the impact of mental health on QoL, and its relationship with the other variables of interest, remains unknown and requires further exploration. Additionally, research considering the specific forms of stigma and social connection may be warranted to further explore the intricacies of these variables. It is also possible that reports of social connectedness and mental health were impacted by the COVID-19 lockdown; as such, a replication study is recommended.

### Implications

The present study provides a novel analysis of the relationship between the variables of interest and how they impact QoL in an Australian context. While existing literature considers the impact of psychosocial variables on QoL, the variables are often considered in isolation; however, these factors often present comorbidly. The mediating effect of social connectedness on the relationship between HIV-related stigma and QoL offers new insights into the complexities of these relationships, providing guidance for clinical intervention.

The findings of the present study were consistent with QoL trends reported in international literature, reflecting that many psychosocial challenges experienced by PLHIV are universal. It is noteworthy that 117 of the 122 participants had achieved VL suppression, highlighting that, despite effective biomedical management, the residual impact of psychosocial comorbidities ultimately detrimentally effects QoL. This supports previous research, suggesting QoL among PLHIV extends beyond traditional medical targets [19]. It is possible, therefore, that QoL among PLHIV in Australia may be more closely linked to psychosocial than biomedical factors [5, 18], strengthening the argument that optimal HIV-management should extend beyond viral suppression. Failing to do so likely ultimately limits the effectiveness of a purely medical healthcare delivery model. The present results support the evolution of HIV-management towards a multidisciplinary model of long-term condition management, whereby psychosocial services play an integral role to holistically optimize QoL.

The results of the present study also demonstrate the importance of specific factors in understanding QoL, providing practitioners with a point of clinical intervention. This is important when considering the Australian Eighth National HIV Strategy, as well as the current NSW: 2021–2025 strategy, which include a QoL target for the first time [35, 61]. This study also strongly supports consideration of the psychosocial determinants of QoL and is consistent with recent Australian Standards for Psychological Support for Adults with HIV [85]. Specifically, interventions to reduce stigma surrounding HIV to improve QoL among PLHIV might be beneficial, in addition to targeting social connectedness to minimize the harmful effects of stigma and improve QoL. There is scope to do this at an organizational level; research suggests that the type of social support (i.e., emotional) is more important than the source of support (e.g., friend versus clinician; [42]), highlighting the important role of mental health practitioners and the therapeutic process itself. Additionally, healthcare services could create avenues to promote connectedness at an individual level, such as optimizing patient supports or implementing peer support programs. Clinical psychosocial interventions could focus on reducing the impact of stigma on mental health, social support, healthcare access and capacity to engage in medical care. This is particularly important when considering the current NSW HIV Strategy: 2021–2025, which explicitly includes a stigma reduction target [32].

## Conclusions

These findings suggest that HIV-related stigma, perceived social connectedness and mental health symptomology are three psychosocial factors that impact QoL among PLHIV in Australia. The results suggest that when these psychosocial factors occur together, the effects of stigma on QoL can operate indirectly through social connectedness. While the present study did not fully capture the relationship of mental health symptomatology to these factors, it is clearly implicated. Collectively, these findings indicate a complex relationship between mental health, stigma, connectedness and QoL, which likely varies across individuals and requires further investigation. By developing this understanding further, practitioners can ensure optimal healthcare delivery and contribute to the expansion of the HIV Cascade beyond viral suppression, to work towards improved wellbeing for PLHIV.

## Data Availability

Not applicable.

## References

[1] Feigin R, Sapir Y, Patinkin N, Turner D (2013). Breaking through the silence: the experience of living with HIV-positive serostatus, and its implications on disclosure. Soc Work Health Care.

[2] Mayer KH (2011). Introduction: Linkage, engagement, and retention in HIV care: essential for optimal individual- and community-level outcomes in the era of highly active antiretroviral therapy. Arch Clin Infect Dis.

[3] Carvalhal A (2010). Are women a different group of HIV-infected individuals?. Arch Women’s Mental Health.

[4] Kwong MK, Ho RT-H, Huang YT (2019). A creative pathway to a meaningful life: an existential expressive arts group therapy for people living with HIV in Hong Kong. Arts Psychother.

[5] Mikołajczak G, Brown G, Power J, Lyons A, Howard C, Drummond F (2020). Social determinants of quality of life among PLHIV in Australia: implications for health promotion. Health Promot Int.

[6] Zeluf-Andersson G, Eriksson L, Schönnesson L, Höijer J, Månehall P, Ekström A (2019). Beyond viral suppression: the quality of life of people living with HIV in Sweden. AIDS Care.

[7] Dessauvagie AS, Jörns-Presentati A, Napp A (2020). The prevalence of mental health problems in sub-Saharan adolescents living with HIV: a systematic review. Glob Ment Health (Camb).

[8] Joint United Nations Programme on HIV/AIDS (UNAIDS). 90–90–90: an ambitious treatment target to help end the AIDS epidemic [Internet]. UNAIDS; October, 2014. https://www.unaids.org/sites/default/files/media_asset/90-90-90_en.pdf?fbclid=IwAR347IPMEqbga2x5d3vw_AZ5SAFvvdaDUQ6wIfzkzKLR16tSje0Mjx5lLUQ

[9] Kay ES, Batey DS, Mugavero MJ (2016). The HIV treatment cascade and care continuum: updates, goals, and recommendations for the future. AIDS Res Ther.

[10] Bulsara SM, Wainberg ML, Rogers K, McAloon J, Grove R, Newton-John TRO (2020). The role of comorbidity on retention in HIV care. AIDS Behav.

[11] Sydney: Kirby Institute. HIV [Internet]. Sydney: Kirby Institute, UNSW Sydney; 2020. https://data.kirby.unsw.edu.au/hiv

[12] World Health Organization. Programme on mental health: WHOQOL user manual [Internet]. Switzerland; 2012. https://www.who.int/publications/i/item/WHO-HIS-HSI-Rev.2012-3

[13] Brown G, Mikołajczak G, Lyons A (2018). Development and validation of PozQoL: a scale to assess quality of life of PLHIV. BMC Public Health.

[14] Nazik E, Arslan S, Nazik H (2013). Determination of quality of life and their perceived social support from family of patients with HIV/AIDS. Sex Disabil.

[15] Hutton VE, Misajon R, Collins FE (2013). Subjective wellbeing and ‘felt’ stigma when living with HIV. Qual Life Res.

[16] Karkashadze E, Gates MA, Chkhartishvili N, Dehovitz J, Tsertsvadze T (2017). Assessment of quality of life in people living with HIV in Georgia. Int J STD AIDS.

[17] Miners A, Phillips A, Kreif N (2014). Health-related quality-of-life of people with HIV in the era of combination antiretroviral treatment: a cross-sectional comparison with the general population. Lancet HIV.

[18] Power J, Amir S, Brown G, et al. HIV futures 9: quality of life among people living with HIV in Australia, monograph series number 116, The Australian Research Centre in Sex, Health and Society, La Trobe University, Melbourne, Australia. 2019.

[19] Lazarus, Safreed-Harmon K, Barton SE (2016). Beyond viral suppression of HIV—the new quality of life frontier. BMC Med.

[20] Guaraldi G, Milic J, Wu A (2019). What is the measure of success in HIV? The fourth 90: quality of life or healthy aging?. Eur Geriatr Med.

[21] HIV Outcomes publishes a set of factsheets on the Health-Related Quality of Life of people living with HIV. HIV Outcomes. 2021. https://hivoutcomes.eu/hiv-outcomes-publishes-a-set-of-factsheets-on-the-health-related-quality-of-life-of-people-living-with-hiv

[22] Adia A, Bermudez A, Callahan M, Hernandez L, Imperial R, Operario D (2018). “An evil lurking behind you”: drivers, experiences, and consequences of HIV–related stigma among men who have sex with men with HIV in Manila Philippines. AIDS Educ Prev.

[23] Flickinger TE, Debolt C, Xie A, Kosmacki A, Grabowski M, Waldman AL (2018). Addressing stigma through a virtual community for people living with HIV: a mixed methods study of the positive links mobile health intervention. AIDS Behav.

[24] Levi-Minzi MA, Surratt HL (2014). HIV stigma among substance abusing people living with HIV/AIDS: implications for HIV treatment. AIDS Patient Care STDS.

[25] Mey A, Plummer D, Dukie S, Rogers GD, O’Sullivan M, Domberelli A (2017). Motivations and barriers to treatment uptake and adherence among people living with HIV in Australia: a mixed-methods systematic review. AIDS Behav.

[26] Nel A, Kagee A (2011). Common mental health problems and antiretroviral therapy adherence. AIDS Care.

[27] Herrmann S, Mckinnon E, Hyland NB (2013). HIV-related stigma and physical symptoms have a persistent influence on health-related quality of life in Australians with HIV infection. Health Qual Life Outcomes.

[28] Logie CH, Wang Y, Lacombe-Duncan A (2018). HIV-related stigma, racial discrimination, and gender discrimination: pathways to physical and mental health-related quality of life among a national cohort of women living with HIV. Prev Med (Baltim).

[29] Andrinopoulos K, Hembling J, Guardado ME, De Maria HF, Nieto AI, Melendez G (2015). Evidence of the negative effect of sexual minority stigma on HIV testing among MSM and transgender women in San Salvador, El Salvador. AIDS Behav.

[30] Ziersch A, Walsh M, Baak M, Rowley G, Oudih E, Mwanri L (2021). “It is not an acceptable disease”: a qualitative study of HIV-related stigma and discrimination and impacts on health and wellbeing for people from ethnically diverse backgrounds in Australia. BMC Public Health.

[31] Earnshaw VA, Chaudoir SR (2009). From conceptualizing to measuring HIV stigma: a review of HIV stigma mechanism measures. AIDS Behav.

[32] Earnshaw VA, Kalichman SC. Stigma experienced by people living with HIV/AIDS. In *Stigma, discrimination and living with HIV/AIDS*. 2013:23–38. Springer, Dordrecht. 10.1007/978-94-007-6324-1_2

[33] Mukherjee A, Lahiry S, Mukherjee A, Choudhury S, Sinha R (2017). Study on defense mechanisms to cope with stress due to stigma among people living with HIV/AIDS reported in Eastern India: a single centre experience. Indian J Clin Med.

[34] Rueda S, Mitra S, Chen S (2016). Examining the associations between HIV-related stigma and health outcomes in people living with HIV/AIDS: a series of meta-analyses. BMJ Open.

[35] Centre for Population health. NSW HIV Strategy 2021–2025 [Internet]. NSW; February 2021, 1–39. https://www.health.nsw.gov.au/endinghiv/Pages/nsw-hiv-strategy-2021-2025.aspx

[36] Slater L, Moneyham L, Vance D, Raper J, Mugavero M, Childs G (2015). The multiple stigma experience and quality of life in older gay men with HIV. J Assoc Nurses AIDS Care.

[37] de Vries DH, Koppen L, Lopez AM, Foppen R (2016). The vicious cycle of stigma and disclosure in "self-management": a study among the Dutch HIV population. AIDS Educ Prev.

[38] Cortes A, Hunt N, Mchale S (2014). Development of the scale of perceived social support in HIV (PSS-HIV). AIDS Behav.

[39] Stafford M, von Wagner C, Perman S, Taylor J, Kuh D, Sheringham J (2018). Social connectedness and engagement in preventive health services: an analysis of data from a prospective cohort study. Lancet Public Health.

[40] Bekele T, Rourke SB, Tucker R (2013). Direct and indirect effects of perceived social support on health-related quality of life in persons living with HIV/AIDS. AIDS Care.

[41] Fredericksen R, Gibbons L, Fitzsimmons E, Crane H (2021). Impact and correlates of sub-optimal social support among patients in HIV care. AIDS Care.

[42] Heywood W, Lyons A (2016). HIV and elevated mental health problems: diagnostic, treatment, and risk patterns for symptoms of depression, anxiety, and stress in a national community-based cohort of gay men living with HIV. AIDS Behav.

[43] Rao D, Chen WT, Pearson CR (2012). Social support mediates the relationship between HIV stigma and depression/quality of life among people living with HIV in Beijing, China. Int J STD AIDS.

[44] Brener L, Broady T, Cama E, Hopwood M, De Wit JBF, Treloar C (2020). The role of social support in moderating the relationship between HIV centrality, internalised stigma and psychological distress for people living with HIV. AIDS Care.

[45] Garrido-Hernansaiz H, Alonso-Tapia J (2017). Social support in newly diagnosed people living with HIV: expectations and satisfaction along time, predictors, and mental health correlates. J Assoc Nurses AIDS Care.

[46] Marziali ME, Mclinden T, Card KG (2021). Social isolation and mortality among people Living with HIV in British Columbia, Canada. AIDS Behav.

[47] Fadzil NA, Othman Z, Mustafa M (2016). Stigma in Malay patients with HIV/AIDS in Malaysia. Int Med J.

[48] Bilardi JE, Hulme-Chambers A, Chen MY (2019). The role of stigma in the acceptance and disclosure of HIV among recently diagnosed men who have sex with men in Australia: a qualitative study. PLoS ONE.

[49] Fekete EM, Williams SL, Skinta MD, Bogusch LM (2016). Gender differences in disclosure concerns and HIV-related quality of life. AIDS Care.

[50] Fekete EM, Williams SL, Skinta MD (2018). Internalised HIV-stigma, loneliness, depressive symptoms and sleep quality in people living with HIV. Psychol Health.

[51] Elopre L, Westfall AO, Mugavero MJ (2016). Predictors of HIV disclosure in infected persons presenting to establish care. AIDS Behav.

[52] Bell S, Aggleton P, Slavin S (2018). Negotiating trust and struggling for control: everyday narratives of unwanted disclosure of HIV status among people with HIV in Australia. Health Sociol Rev.

[53] Stutterheim SE, Brands R, Baas I, Lechner, Kok G, Bos AER (2017). HIV status disclosure in the workplace: positive and stigmatizing experiences of health care workers living with HIV. J Assoc Nurses AIDS Care.

[54] Cama E, Brener L, Slavin S, De Wit J (2017). The relationship between negative responses to HIV status disclosure and psychosocial outcomes among people living with HIV. J Health Psychol.

[55] Remien R, Stirratt M, Nguyen N (2019). Mental health and HIV/AIDS. AIDS.

[56] Eu B, Salleh E, Sakko A, Guaraldi G (2019). Management of human immunodeficiency virus in older people. Aust J Gen Pract..

[57] Lyons A, Pitts M, Grierson J (2012). Exploring the psychological impact of HIV: health comparisons of older Australian HIV-positive and HIV-negative gay men. AIDS Behav.

[58] O'Cleirigh C, Magidson JF, Skeer MR, Mayer KH, Safren SA (2015). Prevalence of psychiatric and substance abuse symptomatology among HIV-infected gay and bisexual men in HIV primary care. Psychosomatics.

[59] Meyer IH (2013). Prejudice, social stress, and mental health in lesbian, gay, and bisexual populations: conceptual issues and research evidence. Psychol Sex Orientat Gend Divers.

[60] Porcelli S, Van Der Wee N, Van Der Werff S (2019). Social brain, social dysfunction and social withdrawal. Neurosci Biobehav Rev.

[61] Department of health. Eighth National HIV Strategy. NSW; 2018. https://www1.health.gov.au/internet/main/publishing.nsf/Content/ohp-bbvs-1/$File/HIV-Eight-Nat-Strategy-2018-22.pdf

[62] Brown G, Mikolajczak G, Lyons A, et al. PozQoL: Valuing quality of life among people with HIV [Internet]. Australian Research Centre in Sex, Health and Society, La Trobe University, Melbourne; 2017. https://www.latrobe.edu.au/__data/assets/pdf_file/0010/843373/PozQoL-Broadsheet.pdf

[63] Engler K, Lessard D, Lebouché B (2017). A review of HIV-specific patient-reported outcome measures. Patient–Patient-centered Outcomes Res.

[64] Wallace DD, Pack A, Uhrig Castonguay B (2019). Validity of social support scales utilized among HIV-infected and HIV-affected populations: a systematic review. AIDS Behav.

[65] Reinius M, Wettergren L, Wiklander M, Svedhem V, Ekström A, Eriksson L (2017). Development of a 12-item short version of the HIV stigma scale. Health Qual Life Outcomes.

[66] Imran N, Afzal H, Aamer I (2020). Scarlett letter: a study based on experience of stigma by COVID-19 patients in quarantine. Pak J Med Sci Q..

[67] American Psychiatric Association. Online assessment measures [Internet]. Washington DC, USA: American Psychiatric Association; 2013. https://www.psychiatry.org/psychiatrists/practice/dsm/educational-resources/assessment-measures

[68] Bravo AJ, Villarosa-Hurlocker MC, Pearson MR (2018). College student mental health: an evaluation of the DSM–5 self-rated Level 1 cross-cutting symptom measure. Psychol Assess.

[69] Gibbons A, Farmer C, Shaw JS, Chung JY (2021). Identifying the factor structure of the DSM-5 level 1 cross-cutting symptom measure. Medrix.

[70] Mahoney M, Farmer C, Sinclair S, Sung S, Dehaut K, Chung J (2020). Utilization of the DSM-5 self-rated level 1 cross-cutting symptom measure-adult to screen healthy volunteers for research studies. Psychiatry Res.

[71] Meaklim H, Swieca J, Junge M (2018). The DSM-5 self-rated level 1 cross-cutting symptom measure identifies high levels of coexistent psychiatric symptomatology in patients referred for insomnia treatment. Nat Sci Sleep.

[72] Online Survey Software - Powering +1B Surveys Annually. [Internet]. Qualtrics; AU, 2022. https://www.qualtrics.com/au/core-xm/survey-software/

[73] Hayes A (2013). Introduction to mediation, moderation, and conditional process analysis.

[74] PozQoL Project. PozQoL Scale Implementation Kit Version 2.0 [Internet]. Australian Research Centre in Sex, Health and Society, La Trobe University, Melbourne; September 2020. http://www.pozqol.org.

[75] Darin-Mattsson A, Fors S, Kåreholt I (2017). Different indicators of socioeconomic status and their relative importance as determinants of health in old age. Int J Equity Health.

[76] Kalan ME, Han J, Taleb ZB (2019). Quality of life and stigma among people living with HIV/AIDS in Iran. HIV AIDS (Auckl).

[77] Reinius M, Rao D, Manhart LE, Wiklander M, Svedhem V, Pryor J (2018). Differential item functioning for items in Berger’s HIV Stigma Scale: an analysis of cohorts from the Indian, Swedish, and US contexts. Qual Life Res.

[78] Zarei N, Joulaei H, Fararouei M (2016). Perceived stigma and quality of life among women living with HIV/AIDS. Womens Health Bull..

[79] Connell J, Brazier J, O’Cathain A, Lloyd-Jones M, Paisley S (2012). Quality of life of people with mental health problems: a synthesis of qualitative research. Health Qual Life Outcomes.

[80] Gooden T, Gardner M, Wang J, Chandan J, Beane A, Haniffa R (2022). The risk of mental illness in people living with HIV in the UK: a propensity score-matched cohort study. Lancet HIV.

[81] Rintamaki LS, Davis TC, Skripkauskas S, Bennett CL, Wolf MS (2006). Social stigma concerns and HIV medication adherence. AIDS Patient Care STDS.

[82] Ekstrand ML, Heylen E, Mazur A (2018). The role of HIV stigma in ART adherence and quality of life among rural women living with HIV in India. AIDS Behav.

[83] Nobre N, Pereira M, Roine RP, Sutinen J, Sintonen H (2018). HIV-related self-stigma and health-related quality of life of people living with HIV in Finland. J Assoc Nurses AIDS Care.

[84] Salfas B, Rendina HJ, Parsons JT (2019). What is the role of the community? Examining minority stress processes among gay and bisexual men. Stigma Health.

[85] Australasian Society for HIV, Viral Hepatitis and Sexual Health Medicine (ASHM). Standards for Psychological Support for Adults with HIV [Internet]. NSW; 2020. https://www.ashm.org.au/resources/hiv-resources-list/australian-standards-psychological-support-adults-hiv/

